# Metabolic Signatures of Kidney *Yang* Deficiency Syndrome and Protective Effects of Two Herbal Extracts in Rats Using GC/TOF MS

**DOI:** 10.1155/2013/540957

**Published:** 2013-09-12

**Authors:** Linjing Zhao, Hongbing Wu, Mingfeng Qiu, Wei Sun, Runmin Wei, Xiaojiao Zheng, Yiting Yang, Xue Xin, Haimiao Zou, Tianlu Chen, Jiajian Liu, Lina Lu, Jing Su, Chungwah Ma, Aihua Zhao, Wei Jia

**Affiliations:** ^1^School of Pharmacy, Shanghai Jiao Tong University, Shanghai 200240, China; ^2^College of Chemistry and Chemical Engineering, Shanghai University of Engineering Science, Shanghai 201620, China; ^3^Infinitus (China) Company Ltd., Guangzhou 510665, China; ^4^Center for Translational Medicine, and Shanghai Key Laboratory of Diabetes Mellitus, Department of Endocrinology and Metabolism, Shanghai Jiao Tong University Affiliated Sixth People's Hospital, Shanghai 200233, China

## Abstract

Kidney *Yang* Deficiency Syndrome (KDS-*Yang*), a typical condition in Chinese medicine, shares similar clinical signs of the glucocorticoid withdrawal syndrome. To date, the underlying mechanism of KDS-*Yang* has been remained unclear, especially at the metabolic level. In this study, we report a metabolomic profiling study on a classical model of KDS-*Yang* in rats induced by hydrocortisone injection to characterize the metabolic transformation using gas chromatography/time-of-flight mass spectrometry. WKY1, a polysaccharide extract from *Astragalus membranaceus* and *Lycium barbarum*, and WKY2, an aqueous extract from a similar formula containing *Astragalus membranaceus*, *Lycium barbarum*, *Morinda officinalis*, *Taraxacum mongolicum*, and *Cinnamomum cassia presl*, were used separately for protective treatments of KDS-*Yang*. The changes of serum metabolic profiles indicated that significant alterations of key metabolic pathways in response to abrupt hydrocortisone perturbation, including decreased energy metabolism (lactic acid, acetylcarnitine), lipid metabolism (free fatty acids, 1-monolinoleoylglycerol, and cholesterol), gut microbiota metabolism (indole-3-propionic acid), biosynthesis of catecholamine (norepinephrine), and elevated alanine metabolism, were attenuated or normalized with different degrees by the pretreatment of WKY1 or WKY2, which is consistent with the observations in which the two herbal agents could ameliorate biochemical markers of serum cortisone, adrenocorticotropic (ACTH), and urine 17-hydroxycorticosteroids (17-OHCS).

## 1. Introduction

The theory of *Yin*-*Yang* is a conceptual framework used for observing and analyzing the material world in ancient China. This philosophical approach has also been adopted for the studies of traditional Chinese medicine (TCM) syndrome and the guidance clinical diagnosis, treatment, and prevention for thousands of years. Kidney *Yang* Deficiency Syndrome (KDS-*Yang*) was firstly documented in Huangdi Neijing, an earliest systematic monograph in TCM. Modern research has shown that damages and functional disorders of hypothalamic-pituitary-target gland axis, including adrenal, thyroid, and gonad, are the main pathological mechanism for forming KDS-*Yang* [1]. Over the past half century, more than 20 experimental models with a variable range of clinical manifestations similar to those observed in KDS-*Yang *human have been developed [2]. It is one of classical methods of establishing KDS-*Yang* to inject animals with high dose of exogenous glucocorticoid, such as hydrocortisone, which would induce adrenocortical insufficiency after abrupt withdrawal administration [2]. This animal model mimicked the pathological state of suppression of hypothalamic-pituitary-adrenal (HPA) axis to some extent in KDS-*Yang* human and contributed greatly to important advances in the current understanding of the underlying mechanisms of KDS-*Yang* as well as treatments [3–5].

Metabolomics, a new omics technique defined as quantitative measurement of time-related multiparametric metabolic response of multicellular systems to pathophysiological stimuli or generic modification [6], has recently developed into an increasingly important tool and has been successfully used in revealing the essence of syndrome and therapeutic effect of TCM. Recently, an increasing number of publications have described the applications of metabolomic approach in evaluating the curative effect and mechanism of traditional medicine in KDS-*Yang* [7–10]. Gong et al. [7] investigated the metabolic profile of hydrocortisone-induced KDS-*Yang* in rats and the intervention effects of *Morinda officinalis *based on nuclear magnetic resonance (NMR). Lu et al. [8] studied the metabonomic characters of KDS-*Yang* rats and the therapeutic effects of *Drynaria fortune* (Kunze) J. Sm. using ultra-performance liquid chromatography coupled with mass spectrometry (UPLC/MS). Li et al. [9] described the serous metabonomic study on *Epimedium brevicornum* Maxim. treated KDS rats and its therapeutic basis using UPLC/MS. Subsequently, an integrated plasma and urinary metabolomics method by UPLC/MS were also developed to reveal the intervention effects of *Epimedium koreanum* Nakai on KDS-*Yang* rats [10]. Also, in our previous study, the urinary metabolic profiles using gas chromatography coupled with mass spectrometry (GC/MS) characterized the biochemical fingerprints of a physiopathologic status similar to KDS in TCM, and the intervention of *Herba Cistanches* could cause a systemic recovery from the hydrocortisone-induced metabolic perturbation in rats [11, 12]. 

WKY1 and WKY2 are two TCM formulas designed for strengthening kidney *Yang*. WKY1 is a crude compound polysaccharide extracted from *Astragalus membranaceus* and *Lycium barbarum*. The combination of these two medicinal plants as a formula is derived from the *Royal Formulary of Yang Spring*, which is part of an ancient health preserving book, “The Life Documentary of Emperor Qianlong.” The rationale for the use of this two-plant formula is to strengthen *Yang* and *Qi* with *Astragalus membranaceus*, while replenishing the *Yin* with *Lycium barbarum* fruits, according to the *Royal Formulary*. The clinical use of the formula is believed to achieve a satisfactory effect of replenishing the kidney deficiency, and therefore, may particularly be effective for chronic colitis and diarrhea generally resulting from the KDS-*Yang*. WKY2 is a mixture of an aqueous extract from a similar Chinese herbal formula, including *Astragalus membranaceus*, *Lycium barbarum*, *Morinda officinalis, Taraxacum mongolicum, and Cinnamomum cassia presl*. The additional three herbs were used in this formula to further strengthen the lung and spleen and thus synergize with the first two herbs to enhance the clinical efficacy of KDS-*Yang*-derived conditions such as chronic diarrhea. Based on our pilot study in dozens of volunteers, the two herbal formulas both showed promising protective effects against KDS-*Yang*.

In this study, a serous metabolic profiling approach based on gas chromatography time-of-flight mass spectrometry (GC/TOF MS) was explored to characterize the metabolic signature of the KDS-*Yang* rats. The protective effects of WKY1 and WKY2 against the metabolic alteration were also investigated with this strategy. The finding of metabolic pathways will be helpful to understand the essence of KDS-*Yang *and the underlying mechanism of the two herbal treatments.

## 2. Materials and Methods

### 2.1. Chemicals and Reagents

HPLC grade methanol, chloroform, and pyridine were purchased from Merck Chemicals (Darmstadt, Germany). Hydrocortisone solution for injection (0.5%) was purchased from Shanghai Xinyi Pharmaceutical Co. (Shanghai, China). *L*-2-chlorophenylalanine was purchased from Intechem Tech. Co. Ltd. (Shanghai, China). BSTFA (1% TMCS), heptadecanoic acid, and methoxyamine were purchased from Sigma Aldrich (St. Louis, MO, USA). All aqueous solutions were prepared with ultrapure water produced by a Milli-Q system (18.2 MΩ, Millipore, Bedford, MA, USA).

### 2.2. Herbal Preparation

The five raw herbs, including *Astragalus membranaceus, Lycium barbarum, Morinda officinalis, Taraxacum mongolicum, and Cinnamomum cassia presl*, were purchased from Chinese mainland and were authenticated on the basis of morphological and chemical analysis in accordance with the Chinese Pharmacopoeia (data not shown). 

WKY1 was produced by water extraction and ethanol precipitation as described previously [13]. In brief, *Astragalus membranaceus* and *Lycium barbarum* mixed at a certain proportion were boiled in 10-fold water for 1.5 h. At the end of 1.5 h boiling, the water extract was collected and the residue was reboiled in 8-fold water for 1 h. The blending supernatant was concentrated in a vacuum rotary evaporator, and then pooled and mixed with ethanol (final concentration 75% v/v) to precipitate the polysaccharide-enriched fraction. The precipitate was separated from the supernatant and vacuum-dried at 40°C to obtain WKY1 (moisture content <7.0%). WKY2 is composed of WKY1 and the powder of an aqueous extract, which was obtained from *Morinda officinalis, Taraxacum mongolicum, and Cinnamomum cassia presl*. For the aqueous extract preparation, three crude herbs were mixed according to proportion and boiled in 10-fold water for 1.5 h. The mixture was cooled to room temperature and filtered. The residue was then refluxed with additional 6-fold water for 1 h. The supernatant was pooled, concentrated under reduced pressure, and then sprayed into dry powder. According to proportion, the spray-drying powder and WKY1 were mixed for obtaining WKY2 (moisture content <5.0%). The total polysaccharide contents of WKY1 and WKY2 were 34.5% (w/w) and 22.6% (w/w), respectively, as determined by the phenol-sulphuric acid method [14]. 

### 2.3. Experimental Design and Sample Collection

The handling of all animals in this study was conformed to the national guidelines and performed at the Center for Laboratory Animals, School of pharmacy, Shanghai Jiao Tong University (SJTU). All the experimental protocols were approved by the Animal Ethics Committee of SJTU. A total of 28 eight-week-old male Sprague-Dawley rats (200 ± 20 g) were commercially obtained from Shanghai Laboratory Animal Co. Ltd. (SLAC, Shanghai, China), housed individually in stainless steel wire mesh cages, and provided with a certified standard rat chow and tap water ad libitum. Room temperature and humidity were regulated at 21 ± 1°C and 60 ± 10%, respectively. A 12/12-h light-dark cycle was set, with lights on at 8 a.m. After 2 weeks of acclimatization in metabolic cages, rats were randomly divided into four groups, 7 in each group: control group (C), which received daily the same volume of saline as the other groups from days 1 to 15; model group (M), which received saline daily from days 1 to 15, then 5% hydrocortisone solution at 50 mg/kg of body weight with i.p. injection from days 16 to 22; WKY1 pretreatment group (WKY1), which received daily WKY1 (30 g/L aqueous suspension) by oral administration at dose of 0.18 g/kg of body weight (equal to 30 times dose of clinic dosage) from days 1 to 15, then 5% hydrocortisone solution daily with i.p. injection at 50 mg/kg from days 16 to 22; and WKY2 pretreatment group (WKY2), which received WKY2 (30 g/L aqueous suspension) at daily oral dose of 1.01 g/kg of body weight (equal to 30 times dose of clinic dosage) from days 1 to 15, then 5% hydrocortisone solution daily at 50 mg/kg with i.p. injection from day 16 to 22. Sera and urine samples were collected at day 25, on the 3rd day after hydrocortisone withdrawal, and stored at −80°C, pending for biochemical and metabolomic analysis. 

### 2.4. Behavioral Observation and Hormone Level Measurement

The weighed chow and water were added into the container of each metabolism cage and the residual food and water were measured daily, respectively. The 24 h urine volume and weekly body weight were recorded, and the general behavior or activity changes of rats were also observed. The serum cortisone, ACTH, and 24 h urine 17-OHCS were measured using ELISA kits (Groundwork Biotechnology Diagnosticate Ltd., San Diego, CA, USA). Data from the serum biochemistry determination were expressed as mean ± SD. Statistical analysis was conducted using the Student's two-tailed, unpaired *t*-test. A *P* value of less than 0.05 was considered statistically significant.

### 2.5. Preparation of Samples and Analysis by GC/TOF MS

Sera samples were derivatized and subsequently analyzed by GC/TOF MS following our previously published protocols [15]. A 100 *μ*L aliquot of serum sample was spiked with two internal standards (10 *μ*L of *L*-2-chlorophenylalanine in water, 0.3 mg/mL; 10 *μ*L of heptadecanoic acid in methanol, 1 mg/mL) and vortexed for 10 s. The mixed solution was extracted with 300 *μ*L of methanol/chloroform (3 : 1) and vortexed for 30 s. After standing for 10 min at −20°C, the samples were centrifuged at 10,000 rpm for 10 min. An aliquot of 300 *μ*L supernatant was transferred to a glass sampling vial to vacuum-dry at room temperature. The residue was derivatized using a two-step procedure. First, 80 *μ*L of methoxyamine (15 mg/mL in pyridine) was added to the vial and kept at 30°C for 90 min followed by 80 *μ*L of BSTFA (1%TMCS) at 70°C for 60 min. Each 1 *μ*L aliquot of the derivatized solution was injected in spitless mode into an Agilent 6890N gas chromatography coupled with a Pegasus HT time-of-flight mass spectrometer (Leco Corporation, St Joseph, MI, USA). Separation was achieved on a DB-5MS capillary column (30 m × 250 *μ*m I.D., 0.25 *μ*m film thickness; (5%-phenyl)-methyl-polysiloxane bonded and crosslinked; Agilent J&W Scientific, Folsom, CA) with helium as the carrier gas at a constant flow rate of 1.0 mL/min. The temperature of injection, transfer interface, and ion source were set to 270°C, 260°C, and 200°C, respectively. The GC temperatures programming was set to 2 min isothermal heating at 80°C, followed by 10°C/min oven temperature ramps to 180°C, 5°C/min to 240°C, and 25°C/min to 290°C, and a final 9 min maintenance at 290°C. Electron impact ionization (70 eV) at full scan mode (*m/z* 30–600) was used, with an acquisition rate of 20 spectrum/second in the TOF MS setting.

### 2.6. Data Processing and Statistical Analysis

The acquired MS files from GC/TOF MS analysis were exported in NetCDF format by ChromaTOF software (v3.30, Leco Co., CA, USA). CDF files were extracted using custom scripts (revised MATLAB toolbox HAD) [15, 16] in the MATLAB 7.1 (The MathWorks, Inc., USA) for data pretreatment procedures such as baseline correction, denoising, smoothing, and alignment; time-window splitting; and peak feature extraction (based on multivariate curve resolution algorithm) [17]. The resulting three-dimensional data set, including sample name, peak retention time, and peak intensity, was imported into the SIMCA-P 11.5 Software package (Umetrics, Umea, Sweden) for data analysis according to our previous publication [18]. Multivariate statistical analysis (partial least-squares-discriminant analysis, PLS-DA) was performed. Meanwhile, significantly expressed features between groups were calculated using a Student's *t*-test (*P* < 0.05). The fold change shows the relative intensity ratio of the differential or representative metabolites between control and model groups with or without WKY1/WKY2 treatments. Additionally, metabolites were annotated by comparing the mass fragments with those of mass spectral in NIST MS databases 2.0 (NIST, Gaithersburg, MD, USA) with a similarity of more than 70% and further verified by the available reference standards. 

## 3. Results and Discussion

### 3.1. Hormone Level and Behavioral Presentation

The blood cortisone, ACTH, and 24 h urine 17-OHCS, which were widely admitted as diadynamic criteria of KDS-*Yang* in clinic of TCM [19], were carried out to assess the success of hydrocortisone-induced KDS-*Yang* model. The results showed that these three hormones levels, as well as body weight, water intake, and 24-h hour urinary excretion, were significantly decreased (*P* < 0.05) in the model group compared to those in the control group on day 25 (Table 1), confirming the suppression of HPA axis and the establishment of KDS-*Yang* model on the 3rd day after hydrocortisone withdrawal (50 mg/kg·d for 7 consecutive days). In addition, the model rats showed the signs of exhaustion, such as reduced activity, idleness, slow response, tending to cluster, and depilate. These pathological changes were in well accordance with those in KDS-*Yang* patients. Pretreatment with WKY1 or WKY2 significantly attenuated the alterations of cortisone and 17-OHCS levels. It was somewhat surprising that the ACTH level significantly increased in the WKY2 group compared with the control group, suggesting that WKY2 might effectively stimulate the anterior pituitary to release ACTH. We also found that the rats in WKY1 and WKY2 treatment groups were more active and curious than the model rats.

### 3.2. Metabolic Variation of KDS-*Yang* Induced by Hydrocortisone

**Figure 1:**
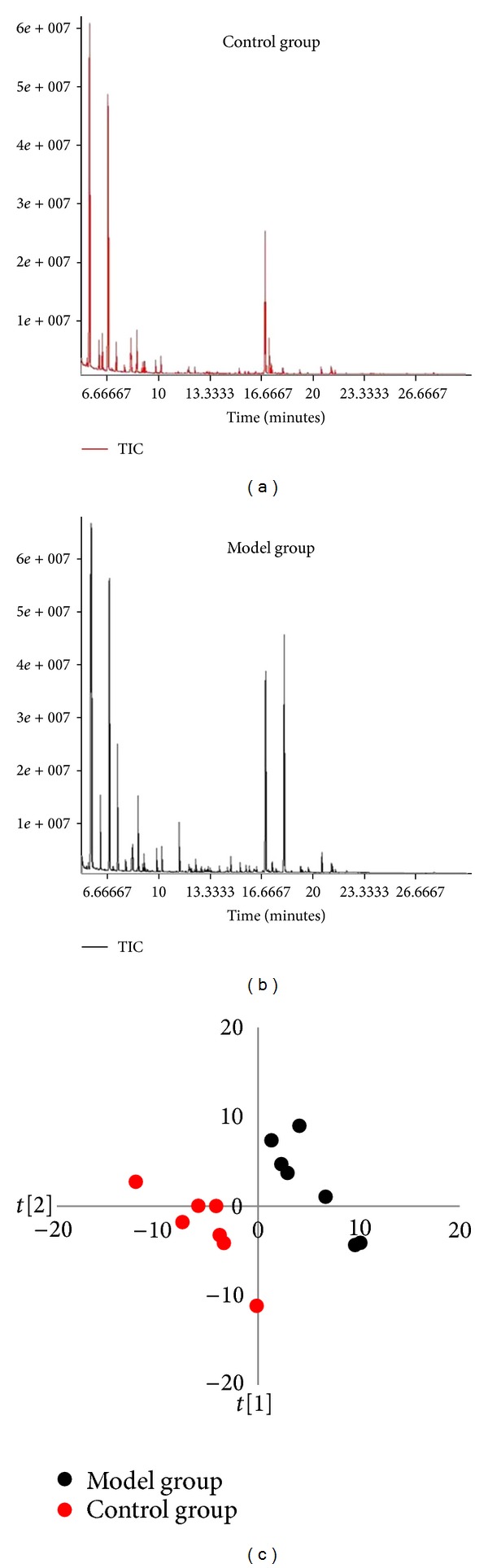
Visualization of biochemical effects of hydrocortisone-induced KDS-*Yang* in rats using a metabolic profiling approach. Typical GC/TOF MS spectra of serum samples from control group (a) and model group (b). (c) Metabolic profiles depicted by PLS-DA sores plot of GC/TOF MS spectral data from serum samples (*n* = 7, each dot denotes an individual rat).

Typical GC/TOF MS total ion current (TIC) chromatograms of serum samples obtained from the control and model rats are illustrated in Figures 1(a) and 1(b). Some obvious differences could be visually inspected between the two groups. A total of 241 peaks were obtained from the GC/TOF MS spectra, and 99 metabolites were identified with NIST 05 standard mass spectral databases with a similarity >70%, further verified by the available reference standards. The multiple pattern recognition PLS-DA scores plot constructed with all the GC/TOF MS data was utilized to depict the general variation between control and model groups (Figure 1(c)). Obvious separations were observed with the stable cumulative modeled variation and good prediction capability with the first two components (component number = 3, *R* ^2^ *X* = 0.420, *R* ^2^ *Y* = 0.993, and *Q* ^2^ = 0.778). The results suggested that the perturbation of serum metabolome occurred in rats on the 3rd day after hydrocortisone withdrawal. The metabolites responsible for the differentiation of metabolic profiles between the two groups were obtained based on a multivariate statistics variable importance in the projection (VIP) threshold of 1.0 from the PLS-DA model. Univariate statistical analysis and Student's *t*-test were performed on metabolites identified from GC/TOF MS analysis of sera samples to evaluate their significance. Fold change was calculated by comparing those metabolites in the model group to the controls. The identified differential metabolites were selected based on the criteria of VIP > 1.0, *P* < 0.05, and fold change (FC) >1.2 or <0.8. A total of 13 differentially expressed metabolites between the control and model groups are listed in Table 2, including decreased lactic acid, acetylcarnitine, glyceraldehyde, histidine, palmitic acid, indole-3-propionic acid, linolic acid, oleic acid, stearic acid, lactose, arachidonic acid, and 1-monolinoleoylglycerol, and increased alanine in the model group compared with the controls. Besides, serum norepinephrine and cholesterol levels tended to decrease in the model group compared to the controls (Table 2).

**Figure 2:**
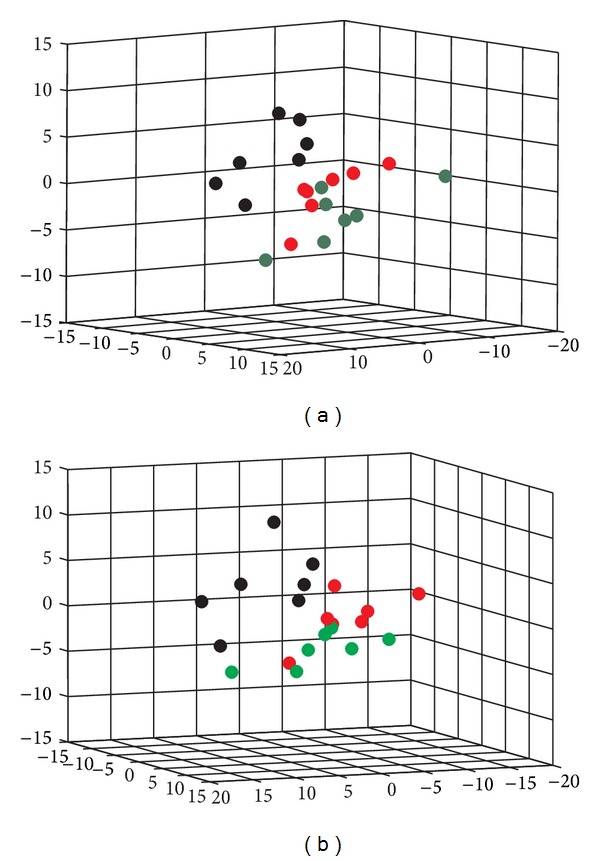
Visualization of biochemical effects of hydrocortisone-induced KDS-*Yang* in rats pretreated with WKY1 (a) and WKY2 (b) using a metabolic profiling approach depicted by a 3D PLS-DA scores plot of GC/TOF MS spectral data from serum samples (*n* = 7; control group, red dot; model group, blank dot; WKY1 treatment group, dark green dots; and WKY2 treatment group, bright green dots; each dot denotes an individual rat).

The metabolic profiling was able to reveal the protective effects of WKY1 or WKY2 on the serum metabolic pattern of KDS-*Yang*. The 3-dimensional PLS-DA scores plots, derived from all variables based on GC/TOF MS spectral data of the control, model, and WKY1 or WKY2 treatment groups on the 3rd day after hydrocortisone withdrawal, are illustrated in Figure 2. The modeling parameters were *R* ^2^ *X*(cum) = 0.510, *R* ^2^ *Y*(cum) = 0.927, *Q* ^2^  (cum) = 0.537 (Figure 2(a)), and *R* ^2^ *X*(cum) = 0.502, *R* ^2^ *Y*(cum) = 0.952, and *Q* ^2^  (cum) = 0.504 (Figure 2(b)), respectively. It could be clearly observed that the two treatment groups were both much closer to the control group than the model group. Among the 15 metabolites responsible for KDS-*Yang *induced by hydrocortisone withdrawal, 13 in WKY1 treatment group except histidine and linolic acid and 14 in WKY2 treatment group except linolic acid had no significant variation (*P* > 0.05) relative to controls on the 3rd day after hydrocortisone withdrawal (Table 2).

**Figure 3:**
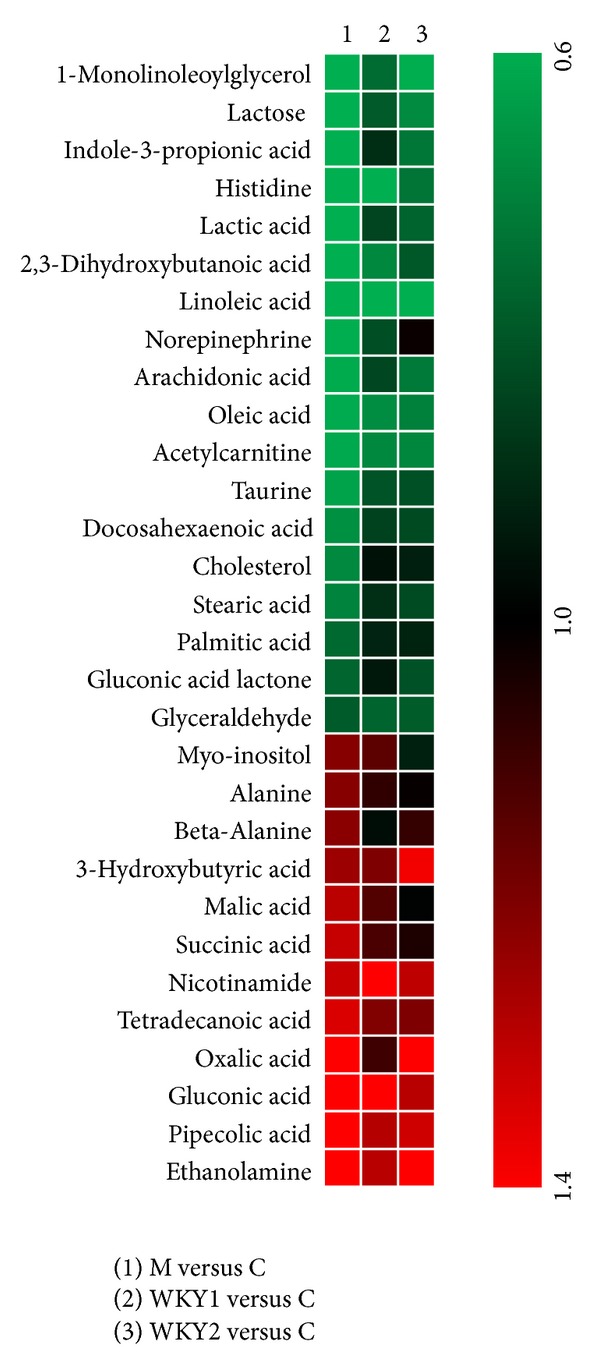
The heatmap of model group; two prophylactic treatment groups (WKY1 and WKY2) compared with controls. Shades of red represent fold increase compared with control, green shades represent fold decrease compared with control, respectively.

We further selected 30 representative metabolites in model rats compared to controls based on the FC and VIP values (FC >1.2 or <0.8 and VIP >1.0). Among these, 16 metabolites in the WKY1 groups, and 16 metabolites in WKY2 group showed significant alterations in FC relative to those in the model group (see Table S1 in Supplementary Material available online at http://dx.doi.org/10.1155/2013/540957). The heatmap generated with the 30 metabolites also indicated less significant fluctuation of metabolite levels in FC relative to controls in both WKY1 and WKY2 groups (Figure 3). To be exact, 17 out of the 30 representative metabolites in WKY1 group and 13 out of the 30 metabolites in WKY2 group were insignificant (in FC, 0.8 < FC < 1.2) relative to controls, as shown in Table S1. These results indicated that pretreatment with WKY1 or WKY2 could effectively attenuate or normalize the metabolic perturbation with different degrees in rats induced by hydrocortisone.

### 3.3. Metabolomic Pathways Associated with KDS-*Yang* and WKY1/WKY2 Treatments

**Figure 4:**
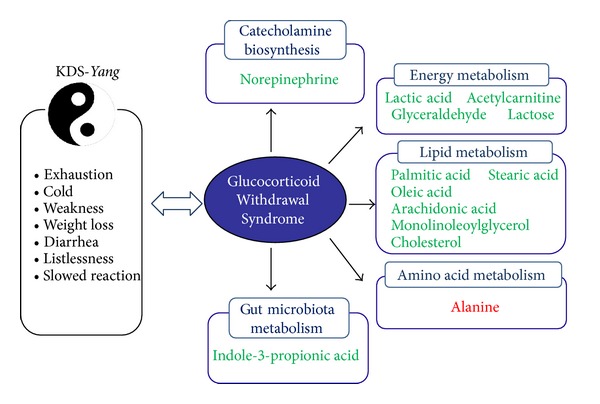
Metabolomic pathways associated with KDS-*Yang* induced by hydrocortisone withdrawal. Green: down-regulated metabolites; Red: up-regulated metabolites.

KDS-*Yang* is one of the elementary syndrome patterns in TCM and closely linked to multiple disordered metabolic pathways. In our study, the model was established through abrupt withdrawal administration after exposure to a high dosage of hydrocortisone for 7 consecutive days in rats. The serum metabolic profiling revealed that the global metabolic perturbation on the 3rd day after hydrocortisone withdrawal, a key time point of KDS-*Yang* study [2], has obviously occurred in model the group characterized by the 15 differentially expressed metabolites listing in Table 2. According to the theory of drugs counterevidence in TCM, most of these metabolites were identified as the important biomarkers associated with KDS-*Yang,* because of their insignificant differential levels in WKY1 or WKY2 pretreatment groups (Table 2). The key biomarkers included lactic acid, acetylcarnitine, glyceraldehyde, lactose, palmitic acid, stearic acid, oleic acid, arachidonic acid, 1-monolinoleoylglycerol, cholesterol, alanine, indole-3-propionic acid, and norepinephrine. Their alterations were associated with multiple metabolic perturbations, including energy metabolism, lipid metabolism, amino acid metabolism, gut microbiota metabolism, and biosynthesis of catecholamine, as summarized in Figure 4. 

Based on the principle of TCM, *Yang* can keep metabolic rate in a normal level to satisfy the normal physiological activities of the human body [20]. In our study, the serum metabolic profiling analysis from KDS-*Yang* rats indicated a significant downregulation in metabolic activities. As illustrated in Table 2, many metabolites related to energy metabolism showed a decreased metabolic tendency in KDS-*Yang* rats compared with controls, such as lactic acid, acetylcarnitine, glyceraldehyde, and lactose. Lactic acid is a predominant source of carbon atoms for glucose synthesis by gluconeogenesis. The regulation of hepatic gluconeogenesis is an important process in the adjustment of the blood glucose level. The serum lactic acid level was observed to significantly decrease statistically in model rats compared with those in the controls, suggesting an inhibitory glycolysis in the KDS-*Yang*. Furthermore, serum acetylcarnitine level in rats exposed to hydrocortisone was significantly decreased relative to controls. Acylcarnitines are synthesized by carnitine acyl transferases from acylCoA and carnitine. Carnitine induces fatty acid *β*-oxidation in the liver [21, 22] and acylcarnitines are substrates for oxidation processes in mitochondria [23]. The decreased level of acetylcarnitine in our study indicated that KDS-*Yang* might be related to the impaired mitochondrial function, compatible with the notion that the lack of glucocorticoids selectively inhibits free fatty acids (FFAs) oxidation [24]. The changes of metabolites were consisted in the state of “exhaustion” in KDS-*Yang* rats, as evidenced by the signs of decreased activity, raritas clothing hair, tendency to cluster, dropped appetite, and weight loss (Table 1). The upregulation of lactate and acetylcarnitine was presented in the two pretreatment groups compared with those in the model group, indicating that WKY1 or WKY2 was able to efficaciously ameliorate the altered energy metabolism and enhance the mitochondria function.

Glucocorticoid excess could cause insulin resistance [25] and cortisol could promote lipolysis [26, 27], rendering it possible that increased levels of FFAs in the circulation may contribute to the observed insulin resistance. Consistently, our data clearly showed that some FFAs, such as palmitic acid, stearic acid, oleic acid, linolic acid, and arachidonic acid, as well as 1-monolinoleoylglycerol significantly decreased in model groups. These results are in agreement with the studies that inhibition of the rise of glucocorticoids could significantly depress plasma FFAs at exhaustion in rodents [28] and that acute cortisol withdrawal could dramatically increase insulin sensitivity in the clinical research [29]. Meanwhile, cholesterol was also found at lower level in model rats. It has been reported that lipoprotein lipase is a key enzyme in the regulation of serum cholesterol, and the activity of the enzyme is increased by glucocorticoids [30]. The decreased cholesterol level in the model group was presumably due to lower activity of lipoprotein lipase, which was caused by the lack of glucocorticoids in KDS-*Yang*. WKY1 and WKY2 effectively attenuated or normalized the alteration of 1-monolinoleoylglycerol, cholesterol, and some FFAs (palmitic acid, stearic acid, oleic acid, and arachidonic acid), suggesting the inhibitory effects of the two herbal medicines on lipid metabolism dysfunction.

According to TCM theory, *Yang* also refers to a cluster of material resources containing excitement and promotion [31]. Our study found that the rats in model group showed the state of lack of vitality, slow reaction, listlessness, and lethargy. It has also been reported that the women with menopausal syndrome of kidney *Yang* deficiency were accompanied by many negative depressive emotions compared with healthy persons [32]. It might be associated with the metabolic dysfunction of some amino acids. In the present study, the serum level of alanine was significantly greater in the model group compared to the controls. It is in agreement with the study that there was a significant positive correlation between the severity of the depression and the plasma levels of alanine [33]. And further, alanine is also an important intermediate regulator in glucose metabolism. Normal blood alanine is transported to liver via glucose-alanine cycle to generate pyruvate which is also an important source for gluconeogenesis [34]. The significantly increased alanine level in model rats indicated that the glucose-alanine cycle was probably impaired in KDS-*Yang*. The normalized expression level of alanine in the WKY1 and WKY2 treatment groups suggests that the two agents' interactions are possibly involved in the glucose-alanine cycle and closely associated with amino acids metabolism.

Chronic diarrhea is another typical signal of KDS-*Yang* patients. However, to date, the pathogenesis has been poorly understood. In our study, the serum level of indole-3-propionic acid (IPA) in rats exposed to hydrocortisone was significantly decreased compared to controls. IPA is a deamination product of tryptophan formed by symbiotic bacteria in the gastrointestinal tract of mammals. The metabolite could be regarded as the characteristic biomarkers of KDS-*Yang* associated with the impaired gut microflora. IPA has been found to protect against oxidative damage caused by carcinogens and other peroxidative agents in animals [35, 36]. The significantly decreased serum IPA level in model rats could help to explain the manifestation of watery diarrhea in rats exposed to hydrocortisone, which was in line with the recent research in which the increased oxidative stress may be responsible for the pathogenesis of diarrhea-related bowel diseases [37]. WKY1 and WKY2 could greatly inhibit the decrease of IPA in serum, suggesting the protective action of WKY1 and WKY2 on gut microbiota metabolism.

In the present study, the decreased serum norepinephrine level in KDS-*Yang* rats was observed. Norepinephrine is an important neurotransmitter secreted by the adrenal medulla. The decreased serum norepinephrine could ascribe to the downregulation of catecholamine biosynthetic pathway in KDS-*Yang* status. The result was compatible with the previous experiments in which the levels of norepinephrine in the blood of patients with *Yang* hyperactivity syndrome were higher than those of healthy people [38]. So, it is believed that the sympathetic nervous system suppression is an important pathological progress of KDS-*Yang*, and the levels of norepinephrine might be thought to be a vitally important diagnostic biomarker for KDS-*Yang*. The normalized expression level of norepinephrine in the two treatment groups suggests the protective effect of WKY1 and WKY2 on catecholamine pathways.

Our results revealed that the interventions in energy metabolism, lipid, amino acid, gut microbiota metabolism, and biosynthesis of catecholamine might be the critical factors for WKY1 or WKY2 to prevent the experimental KDS-*Yang* rats suffering from the disorder of metabolism. And further, some differences were shown between the two prophylactic treatment groups. To be exact, lactose, lactic acid, 1-monolinoleoylglycerol, stearic acid, arachidonic acid, cholesterol, and IPA levels in sera samples of WKY1 group appeared more similar as normal, while the serum levels of alanine and norepinephrine in WKY2 group were much closer to control levels than those in WKY1 group (Figure 3; Table S1). The results suggested that the preventive effect of WKY1, the crude compound polysaccharides obtained from *Astragalus membranaceus* and *Lycium barbarum, *was superior to the aqueous extract of *Morinda officinalis, Taraxacum mongolicum,*  and  *Cinnamomum cassia presl *on energy metabolism, lipid metabolism, and gut microbiota metabolism. And, WKY2 was more effective on amino acid metabolism and catecholamine pathways than WKY1, possibly related to the aqueous extract of three additional herbs. Further studies of the two original herbal agents are necessary to develop plant-derived therapeutic medicines for the prevention and treatment of the metabolic dysfunction from KDS-*Yang*.

## 4. Conclusion

In the paper, a serum metabolomic profiling approach based on GC/TOF MS has been developed to investigate the specific physiopathologic state of KDS-*Yang* in rats induced by hydrocortisone withdrawal. Some potential biomarkers, such as lactic acid, acetylcarnitine, some FFAs, alanine, indole-3-propionic acid, and norepinephrine, were found and identified. The metabolic shifts of these metabolites could help explain the clinical manifestation of KDS-*Yang* and further reveal the alterations in energy metabolism, lipid, amino acid, gut microbiota metabolism, and biosynthesis of catecholamine induced by hydrocortisone. Two innovative herbal agents of WKY1 and WKY2 could attenuate or normalize the metabolic transformation with different degrees on these metabolic pathways.

## Supplementary Material

The changes in the levels of 30 representative metabolites indicated that pretreatment with WKY1 or WKY2 could effectively attenuate or normalize the metabolic perturbation with different degrees in rats induced by hydrocortisone.”

## Figures and Tables

**Table 1:** Hormone variation and behavioral investigation results on the 3rd day after hydrocortisone withdrawal with or without WKY1/WKY2 pretreatments.

Groups	Cortisone (ng/mL)	ACTH (pg/mL)	17-OHCS (*μ*mol/L)	Body weight (g)	Food consumption (g)	Water intake (mL)	Urine volume (mL)
Control group	220.78 ± 105.78	633.43 ± 111.1	433.81 ± 15.64	371.9 ± 7.2	14.8 ± 1.3	48.9 ± 4.3	40.6 ± 5.9
Model group	139.21 ± 40.79**	447.14 ± 126.5*	398.91 ± 29.13**	349.0 ± 12.0**	13.4 ± 1.1	36.3 ± 4.5**	18.4 ± 6.0**
WKY1 group	161.84 ± 87.27	632.98 ± 50.65	400.6 ± 16.59	356.6 ± 15.6*	15.1 ± 2.5	42.3 ± 5.9*	27.9 ± 8.4**
WKY2 group	206.51 ± 71.64	993.26 ± 17.46**	416.79 ± 12.78	357.4 ± 12.4*	14.9 ± 2.1	34.8 ± 4.6**	20.4 ± 2.8**

*P < 0.05, **P < 0.01 compared with control group (two-tailed Student's *t*-test).

**Table 2:** List of differential serum metabolites in M group on the 3rd day after hydrocortisone withdrawal, and *P* values in M, WKY1, and WKY2 groups compared to controls.

Metabolites	Formula	RT (min)	VIP^a^	FC^b^	*P* ^c^		
					M	WKY1	WKY2
Lactic acid	C_3_H_6_O_3_	5.54	1.43	0.58	0.043	0.545	0.312
Alanine	C_3_H_7_NO_2_	6.19	1.40	1.21	0.036	0.535	0.916
Acetylcarnitine	C_9_H_17_NO_4_	6.39	1.42	0.62	0.034	0.172	0.076
Glyceraldehyde	C_3_H_6_O_3_	6.77	1.46	0.80	0.033	0.085	0.080
Histidine	C_6_H_9_N_3_O_2_	17.29	1.83	0.58	0.002	0.004	0.050
Palmitic acid	C_16_H_32_O_2_	19.16	1.35	0.76	0.047	0.450	0.603
Indole-3-propionic acid	C_11_H_11_NO_2_	19.63	1.37	0.56	0.038	0.676	0.303
Linoleic acid	C_18_H_32_O_2_	21.18	1.46	0.61	0.021	0.009	0.007
Oleic acid	C_18_H_34_O_2_	21.23	1.37	0.61	0.035	0.070	0.139
Stearic acid	C_18_H_36_O_2_	21.42	1.63	0.70	0.008	0.448	0.162
Arachidonic acid	C_20_H_32_O_2_	22.15	1.47	0.61	0.024	0.382	0.113
1-Monolinoleoylglycerol	C_21_H_38_O_4_	22.36	1.71	0.23	0.004	0.556	0.158
Norepinephrine	C_8_H_11_NO_3_	22.61	1.28	0.61	0.065	0.417	0.947
Lactose	C_12_H_22_O_11_	23.63	1.48	0.50	0.027	0.433	0.114
Cholesterol	C_27_H_46_O	27.85	1.33	0.69	0.066	0.811	0.716

^a^VIP was obtained from PLS-DA model (Figure 1(c)).

^b^FC with a value >1 indicates a relatively higher concentration while a value <1 means a relatively lower concentration present in M group as compared to the controls.

^c^ *P* value of Student's *t* test.

## Abbreviations

17-OHCS: 17-hydroxycorticosteroids
ACTH: Adrenocorticotropic hormone
FFAs: Free fatty acids
GC/MS: Gas chromatography coupled with mass spectrometry
GC/TOF MS: Gas chromatography coupled with time-of-flight mass spectrometry
HC: Hydrocortisone
HPA axis: Hypothalamic-pituitary-adrenal axis
IPA: Indole-3-propionic acid
KDS-*Yang*: Kidney-*Yang* Deficiency Syndrome
NMR: Nuclear magnetic resonance
PLS-DA: Partial least squares discriminant analysis
TCM: Traditional Chinese medicine
UPLC/MS: Ultra Performance liquid chromatography coupled with mass spectrometry.
