# Supplementary material for: Metabolic Signatures of Kidney Yang Deficiency Syndrome and Protective Effects of Two Herbal Extracts in Rats Using GC/TOF MS
Source: Evid Based Complement Alternat Med. 2013 Sep 12;2013:540957. doi: 10.1155/2013/540957 (PMC3789486; doi:10.1155/2013/540957)
Supplement: Supplementary file 1 — The changes in the levels of 30 representative metabolites indicated that pretreatment with WKY1 or WKY2 could effectively attenuate or normalize the metabolic perturbation with different degrees in rats induced by hydrocortisone.” [file 540957.f1.docx]

**Supporting Information**

The changes in the levels of 30 representative metabolites indicated that pretreatment with WKY1 or WKY2 could effectively attenuate or normalize the metabolic perturbation with different degrees in rats induced by hydrocortisone.

Table S1. List of identiﬁed representative metabolites in serum on the 3rd day after hydrocortisone withdrawal, and FC of WKY1 and WKY2 groups relative to control and model respectively.

| NO. | Metabolites^a)^ | HC Exposure | |  | WKY1 Treatment | |  | WKY2 Treatment | |
| --- | --- | --- | --- | --- | --- | --- | --- | --- | --- |
|  |  | VIP | M vs. C |  | WKY1 vs. C^b)^ | WKY1 vs. M^c)^ |  | WKY2 vs. C^b)^ | WKY2 vs. M^c)^ |
| 1 | 1-Monolinoleoylglycerol | 1.71 | 0.230 |  | 0.755 | **3.278** |  | 0.608 | **2.639** |
| 2 | Lactose | 1.48 | 0.501 |  | 0.797 | **1.591** |  | 0.685 | **1.367** |
| 3 | Indole-3-propionic acid | 1.37 | 0.563 |  | **0.897** | **1.594** |  | 0.730 | **1.297** |
| 4 | Histidine | 1.83 | 0.576 |  | 0.581 | 1.010 |  | 0.735 | **1.277** |
| 5 | Lactic acid | 1.43 | 0.576 |  | **0.845** | **1.467** |  | 0.774 | **1.344** |
| 6 | 2,3-Dihydroxybutanoic acid | 1.14 | 0.587 |  | 0.692 | 1.180 |  | **0.803** | **1.368** |
| 7 | Linolic acid | 1.46 | 0.605 |  | 0.562 | 0.930 |  | 0.556 | 0.919 |
| 8 | Norepinephrine | 1.28 | 0.606 |  | **0.823** | **1.359** |  | **1.017** | **1.679** |
| 9 | Arachidonic acid | 1.47 | 0.613 |  | **0.839** | **1.369** |  | 0.724 | 1.181 |
| 10 | Oleic acid | 1.37 | 0.614 |  | 0.680 | 1.108 |  | 0.709 | 1.155 |
| 11 | Acetylcarnitine | 1.42 | 0.617 |  | 0.690 | 1.119 |  | 0.694 | 1.126 |
| 12 | Taurine | 1.21 | 0.631 |  | **0.812** | **1.286** |  | **0.819** | **1.298** |
| 13 | Decosahexaenoic acid | 1.21 | 0.674 |  | **0.852** | **1.263** |  | **0.833** | **1.236** |
| 14 | Cholesterol | 1.33 | 0.690 |  | **0.960** | **1.392** |  | **0.930** | **1.349** |
| 15 | Stearic acid | 1.63 | 0.702 |  | **0.894** | **1.273** |  | **0.831** | 1.183 |
| 16 | Palmitic acid | 1.35 | 0.762 |  | **0.919** | **1.206** |  | **0.921** | **1.208** |
| 17 | Gluconic acid lactone | 1.40 | 0.772 |  | **0.947** | **1.226** |  | **0.815** | 1.055 |
| 18 | Glyceraldehyde | 1.46 | 0.795 |  | 0.774 | 0.974 |  | 0.791 | 0.995 |
| 19 | Myo-inositol | 1.34 | 1.211 |  | **1.146** | 0.946 |  | **0.928** | **0.766** |
| 20 | Alanine | 1.40 | 1.212 |  | **1.077** | 0.888 |  | **1.011** | 0.834 |
| 21 | Beta-Alanine | 1.12 | 1.217 |  | **0.971** | **0.798** |  | **1.082** | 0.889 |
| 22 | 3-Hydroxybutyric acid | 1.10 | 1.246 |  | **1.199** | 0.963 |  | 1.382 | 1.109 |
| 23 | Malic acid | 1.30 | 1.294 |  | **1.129** | 0.873 |  | **0.994** | **0.768** |
| 24 | Succinic acid | 1.42 | 1.311 |  | **1.117** | 0.852 |  | **1.046** | **0.797** |
| 25 | Nicotinamide | 1.15 | 1.315 |  | 1.639 | 1.247 |  | 1.301 | 0.990 |
| 26 | Tetradecanoic acid | 1.06 | 1.342 |  | 1.203 | 0.896 |  | 1.200 | 0.894 |
| 27 | Oxalic acid | 1.09 | 1.405 |  | **1.099** | **0.782** |  | 1.539 | 1.095 |
| 28 | Gluconic acid | 1.78 | 1.493 |  | 1.596 | 1.069 |  | 1.290 | 0.864 |
| 29 | Pipecolinic acid | 1.14 | 1.940 |  | 1.284 | **0.662** |  | 1.325 | **0.683** |
| 30 | Ethanolamine | 1.29 | 2.234 |  | 1.291 | **0.578** |  | 1.612 | **0.721** |

^a)^ The metabolites were selected in model rats compared to controls based on the FC and VIP values (FC>1.2 or <0.8 and VIP>1.0)

^b)^ Bold data means the metabolites were insignificant in FC (0.8<FC<1.2) relative to controls.

^c)^ Bold data means the metabolites were signiﬁcantly altered in FC (>1.2 or <0.8) relative to model group.
